# In Vitro Fermentation Behavior of Isomalto/Malto‐Polysaccharides Using Human Fecal Inoculum Indicates Prebiotic Potential

**DOI:** 10.1002/mnfr.201800232

**Published:** 2018-05-28

**Authors:** Fangjie Gu, Klaudyna Borewicz, Bernadette Richter, Pieter H. van der Zaal, Hauke Smidt, Pieter L. Buwalda, Henk A. Schols

**Affiliations:** ^1^ Laboratory of Food Chemistry Wageningen University & Research P.O. Box 17 6700 AA Wageningen The Netherlands; ^2^ Laboratory of Microbiology Wageningen University & Research Wageningen 6708 WE The Netherlands; ^3^ Biobased Chemistry & Technology Wageningen University & Research Wageningen 6708 WG The Netherlands; ^4^ Coöperatie AVEBE U.A. P.O. Box 15 9640 AA Veendam The Netherlands

**Keywords:** digesta, enzyme activities, microbiota, short chain fatty acids

## Abstract

**Scope:**

This study characterize intestinal fermentation of isomalto/malto‐polysaccharides (IMMPs), by monitoring degradation of IMMPs, production of short chain fatty acids (SCFAs), lactic acid, and succinic acid as well as enzyme activity and microbiota composition.

**Methods and results:**

IMMP‐94 (94% α‐(1→6) glycosidic linkages), IMMP‐96, IMMP‐27, and IMMP‐dig27 (IMMP‐27 after removal of digestible starch segments) are fermented batchwise in vitro using human fecal inoculum. Fermentation digesta samples are taken for analysis in time up till 48 h. The fermentation of α‐(1→6) glycosidic linkages in IMMP‐94, IMMP‐96, and IMMP‐dig27 starts after 12 h and finishes within 48 h. IMMP‐27 fermentation starts directly after inoculation utilizing α‐(1→4) linked glucosyl residues; however, the utilization of α‐(1→6) linked glucoses is delayed and start only after the depletion of α‐(1→4) linked glucose moieties. SCFAs are produced in high amounts with acetic acid and succinic acid being the major products next to propionic acid and butyric acid. The polysaccharide fraction is degraded into isomalto‐oligosaccharides (IMOs) mainly by extracellular enzymes. The smaller IMOs are further degraded by cell‐associated enzymes. Overall microbial diversity and the relative abundance of *Bifidobacterium* and *Lactobacillus*, significantly increase during the fermentation of IMMPs.

**Conclusion:**

IMMP containing segments of α‐(1→6) linked glucose units are slowly fermentable fibers with prebiotic potential.

## 1 Introduction

Prebiotics and their health benefits are of growing research interest nowadays. A dietary prebiotic is defined as “a substrate that is selectively utilized by host microorganisms conferring a health benefit.”^1^ Well‐documented prebiotics include lactulose, inulin, fructo‐oligosaccharides (FOS), and galacto‐oligosaccharides (GOS). These substrates have been shown to selectively stimulate the growth and activity of bifidobacteria, lactic acid bacteria, and other health beneficial bacteria.^2^, ^3^ Fermentation of prebiotics in the colon by these and other bacterial groups leads to the production of short chain fatty acids (SCFAs) that are beneficial for gut health.^4^ The present study focuses on a novel type of undigestible α‐glucans, the isomalto/malto‐polysaccharides (IMMPs).

IMMPs are produced from starch with the use of a 4,6‐α‐glucanotransferase (GTFB) enzyme from *Lactobacillus reuteri* 121.^5^, ^6^ The GTFB enzyme transfers a glucose moiety from the nonreducing end of α‐(1→4) linked glucose chains, as present in starch and starch‐derived maltodextrins, to the nonreducing end of other glucose chain generating α‐(1→6) linkages between glucose units in a stepwise manner, which results in the formation of IMMPs containing linear chains of α‐(1→6) linked glycose residues.^6^ The conversion rate to α‐(1→6) linkages is positively correlated with the amylose content of the substrates, and negatively correlated with the original level of α‐(1→4,6) linked glucose moieties present in amylopectin.^6^ For this reason, the joint action of GTFB and debranching enzymes, for example, isoamylase or pullulanase, leads to higher conversion rates from α‐(1→4) to α‐(1→6) glycosidic linkages.^6^ The percentage of α‐(1→6) glycosidic linkages can reach more than 90%, depending on the origin of the starch used as the substrate and the involvement of debranching enzymes.^6^

IMMPs have been suggested to have potential health beneficial effects because the α‐(1→6) rich segments can escape digestion in the upper gastrointestinal tract, and be utilized as carbon source by microbiota in the large intestine.^6^ This has been reported for compounds such as isomalto‐oligosaccharides (IMOs) and dextran, which have similarities in structure when compared to IMMPs. IMOs are gluco‐oligosaccharides consisting of predominantly α‐(1→6) linkages, with the degree of polymerization (DP) ranging from 2 to 10.^7^ IMOs have been shown to promote the growth of lactobacilli and bifidobacteria in both in vitro fermentation and in vivo rat models.^8^, ^9^, ^10^ Dextran, another well‐known glucose homopolysaccharide with consecutive α‐(1→6) linkages, has been reported to stimulate bifidobacteria and lactobacilli during in vitro fermentation with human fecal microbiota, and to lead to increased production of butyrate.^11^ Therefore, based on the structural similarity between IMMPs, IMOs, and dextran, we expected that IMMPs would bear prebiotic potential as well.

The starch origin and involvement of debranching enzymes during the synthesis of IMMPs lead to structural differences, which in turn, may influence the IMMPs’ fate during fermentation in the colon. The difference can be in the proportion of α‐(1→6) and α‐(1→4) glycosidic linkages. It has been shown by NMR spectroscopy that the relative amount of α‐(1→6) linkages can be very different, ranging from 7% to over 90%,^6^ with the remaining linkages being α‐(1→4). Although α‐(1→4) linkages are in general readily digested by human digestive enzymes, introduction of α‐(1→6) linkages may help neighboring α‐(1→4) linked units to escape digestion and to enter the colon. Such starches that have been chemically or enzymatically modified to resist digestion, are considered to be resistant starch type IV.^12^ It remains unclear to what extent the α‐(1→4) linked glucose segments of IMMPs would end up in the colon and have an influence on fermentation of α‐(1→6) linked glucosyl residues. Based on earlier studies on the fate of retrograded tapioca starch, it can be speculated that the presence of resistant starch could influence the fermentation of other fibers.^13^ IMMPs with similar percentages of α‐(1→6) linkages could differ in the distribution of molecular chain length, depending on the side‐chain length distribution of the parental starch. It remains unknown whether such differences in molecular chain length would influence the fermentation behavior of IMMPs.

Leemhuis et al.^6^ showed preliminary results of in vitro fermentation of IMMPs, including an increase in microbial biomass, as monitored by optical density, and an increase in concentrations of acetic acid and propionic acid. However, the influence of additional factors on IMMPs fermentation, including molecular weight and the presence of α‐(1→4) linkages, still needs to be determined. Furthermore, previous research showed that the production of enzymes by fecal microbiota varies depending on substrate properties, including sugar composition, linkage type, and chain length.^13^, ^14^, ^15^ The prebiotic potential of IMMPs is still unknown since detailed effects on microbiota composition are yet to be established.^6^

Therefore, to evaluate the prebiotic potential of IMMPs, a comprehensive in vitro batch fermentation of selected types of IMMPs with a standardized human fecal inoculum was performed in the present study. The fermentation behavior of IMMPs at a molecular level and the production of individual organic acids were studied, and a link to microbiota composition was made. In addition, bacterial enzyme activities involved in the IMMP degradation were studied in order to help explaining the mechanism of bacterial utilization of IMMPs.

## 2 Experimental Section

### 2.1 Materials

Three different types of IMMPs were used in this study. In order to facilitate the comparison of results, the IMMPs in this study were named after their percentages of total α‐(1→6) glucosyl linkages. The total α‐(1→6) linked glucosyl content, consisting of both α‐(1→6) and α‐(1→4,6) linked glucosyl residues, was determined by hydrogen‐1 nuclear magnetic resonance (^1^H NMR) spectroscopy, with the methodology and results already published previously.^16^ IMMP‐94 (94% α‐(1→6) linkages) originates from potato starch (AVEBE, Veendam, The Netherlands) modified with *L. reuteri* 121 GTFB 4,6‐α‐glucanotransferase^6^ and pullulanase (Promozyme D2; Novozymes, Bagsvaerd, Denmark) and was kindly provided by Dr. Hans Leemhuis (AVEBE). IMMP‐27 (27%) and IMMP‐96 (96%) were synthesized from potato starch and Etenia 457 starch (AVEBE), respectively, as published by van der Zaal et al.^16^ and described below.

#### 2.1.1 IMMP‐27

Potato starch was suspended at 2.5% w/v in 20 mM sodium acetate buffer, pH = 4.9, containing 5 mM CaCl_2_. The suspension was autoclaved at 121 °C for 15 min and cooled to 37 °C. IMMP synthesis was carried out by adding 0.3 mg GTFB‐ΔN per gram substrate and incubating the reaction mixture at 37 °C for 24 h. GTFB‐ΔN is GTFB with N‐terminal truncation,^17^ and the synthesis of GTFB‐ΔN is described elsewhere.^16^ GTFB‐ΔN was inactivated in a water bath at 95 °C for 15 min. The solution was cooled to 50 °C; AMBERLITE MB‐20 Resin (Dow, Midland, MI, USA) was added to remove salts and then incubated at 50 °C for 2 h. The resin was sieved out and the IMMP solution was freeze‐dried.

#### 2.1.2 IMMP‐96

Amylomaltase‐treated potato starch (Etenia 457) was used as substrate, and treated similarly as described for the synthesis of IMMP‐27, with some modifications. Besides GTFB‐ΔN, pullulanase (Promozyme D2) was also added at an amount of 2 μL g^−1^ substrate, and the incubation time was extended to 41 h.

### 2.2 Experimental Setup and Removal of Digestible Starch Segments

**Figure 1:**
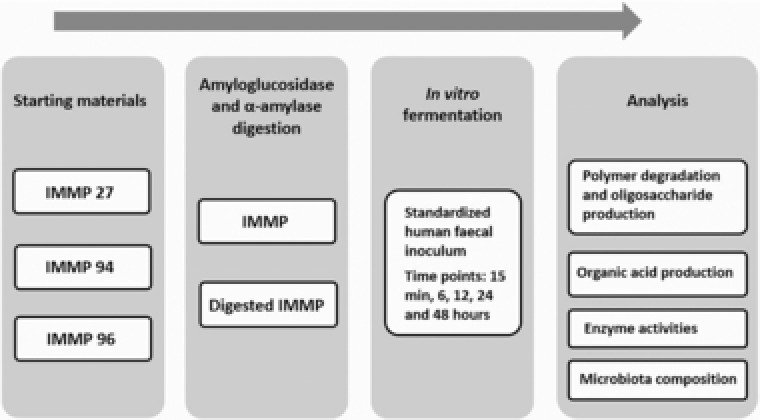
Experimental setup of the in vitro fermentation of IMMPs.

A schematic overview of the present study is shown in **Figure** 1. Three types of IMMPs, IMMP‐27, IMMP‐94, and IMMP‐96, were used representing extremes with respect to the percentage of total α‐(1→6) linked glucosyl residues. Each of the three IMMPs were split and parts were either left untreated or treated with pancreatic α‐amylase and amyloglucosidase (Resistant Starch Assay Kit, Megazyme, Bray, Ireland) to remove α‐(1→4) linked glucosyl residues in order to obtain the resistant fiber. Hereto, IMMP‐27, IMMP‐94, and IMMP‐96 were treated with two starch digesting enzymes. The concentrations of both enzymes and the incubation conditions were according to Megazyme protocols. After inactivating the enzymes at 100 °C for 5 min, IMMPs were recovered by ethanol precipitation with a final ethanol concentration of 70%. The supernatant containing glucose and small maltodextrins was removed by decanting after centrifugation at 10 000 × *g* for 15 min at room temperature. The ethanol precipitation step was repeated twice. Afterward, the pellet was washed once with pure ethanol and air‐dried at 30 °C. The sample obtained after the removal of digestible α‐1,4‐linked glucose segments from IMMP‐27 was named IMMP‐dig27.

In order to determine the level of removal of α‐(1→4) linked glucosyl residues in IMMPs after enzymatic digestion, the sugar content of the supernatant collected from the ethanol precipitation step was measured colorimetrically by a phenol‐sulfuric acid assay, using d‐glucose as a standard for calibration.^18^, ^19^ Since less than 2% of glucose moieties were removed from both IMMP‐94 and IMMP‐96, only the parental IMMP‐94 and IMMP‐96 were included in the following in vitro fermentation, whereas both untreated IMMP‐27 and IMMP‐dig27 were used.

#### 2.2.1 In Vitro Fermentation

An in vitro fermentation was performed to simulate the fermentation of IMMPs in the human colon according to the procedure described by Rösch et al.,^14^ with the modification that carbohydrates (pectin, xylan, arabinogalactan, amylopectin, and starch) and Tween 80 were left out of the standard ileal efflux medium (SIEM), in order to reduce as much as possible background fermentation from the medium components. The modified SIEM medium contained 40% v/v BCO medium, 1.6% v/v salt solution, 0.8% v/v MgSO_4_ (50 g L^−1^), 0.4% v/v cysteine hydrochloride (40 g L^−1^), 0.08% v/v vitamin solution, and 10% v/v MES buffer (1 M, pH 6.0) in water. The BCO medium contained (g L^−1^): Bacto Peptone, 60.0; casein, 60.0; Ox Bile, 1. The salt solution contained (g L^−1^): K_2_HPO_4_·3H_2_O, 156.25; NaCl, 281.25; CaCl_2_·2H_2_O, 28.13; FeSO_4_·7H_2_O, 0.31; hemin, 0.63. The vitamin solution contained (mg L^−1^): menadione, 1.0; biotine, 2.0; vitamin B12, 0.5; pantothenate, 10.0; nicotinamide, 5.0; *p*‐aminobenzoic acid, 5.0; thiamine, 4.0. The ingredients used to make SIEM were purchased from Tritium Microbiologie (Veldhoven, The Netherlands).

A standard human fecal inoculum was prepared by TNO (Zeist, The Netherlands), and was kindly provided by Prof. Dr. K. Venema. The fecal inoculum was pooled from seven healthy volunteers (male: *n* = 3, average age = 46.3 years [range: 26–57 years], BMI = 24.1 ± 2.42 kg m^−2^; female: *n* = 4, average age = 37.7 years [27–52 years], BMI = 24.2 ± 1.91 kg m^−2^). The pooling procedure was described and validated previously.^20^, ^21^

Each in vitro fermentation took place in a 20 mL serum bottle sealed with a butyl rubber stopper and with the final volume of the fermentation liquid being 10 mL. The final concentration of IMMP was 10 mg mL^−1^ and fecal inoculum was added to a final concentration of 1% v/v. The fermentation was performed in duplicate. All handling procedures were performed in an anaerobic cabinet (gas phase: 81% N_2_, 15% CO_2_, and 4% H_2_). Negative control incubations were included and did not receive any fecal inoculum (inoculum blanks) or IMMP substrate (IMMP blanks). A baseline sample (defined as 0 h) was taken within the first 15 min after the addition of the inoculum, after which bottles were incubated at 37 °C and shaked at 140 rpm. For sampling, sterile syringes and needles were used to take aliquots (2–3.5 mL) at time points 15 min, 6 h, 12 h, 24 h, and 48 h.

### 2.3 Analytical Methods

#### 2.3.1 Assessment of IMMP Degradation by HPSEC‐RI

Part of the fermentation digest was heated at 100 °C for 5 min and then centrifuged at 18 600 × *g* for 10 min at room temperature. The supernatant was diluted four times with water to be used for high performance size exclusion chromatography (HPSEC). A set of four TSK‐Gel SuperAW columns (Tosoh Bioscience, Tokyo, Japan) were used on an Ultimate 3000 HPLC (Dionex, Sunnyvale, CA, USA) system: a guard column (SuperAW‐L, 3.5 cm × 4.6 mm ID) and three analytical columns (SuperAW 4000, 3000, and 2500; 15 cm × 6.0 mm ID). Ten μL of sample was injected and eluted at 0.6 mL min^−1^ 0.2 M NaNO_3_ isocratically. The column temperature was 55 °C. Eluted components were monitored by an RI detector (Shodex RI‐101; Showa Denko K.K., Kawasaki, Japan). Molecular weights of IMMPs were estimated using a pullulan (Polymer Laboratories, Palo Alto, CA, USA) calibration curve. Chromeleon 7.1 software (Dionex) was used to process data from HPSEC.

#### 2.3.2 Analysis of Oligosaccharide Production by HPAEC‐PAD

The supernatant obtained after centrifugation of the fermentation digest was tenfold diluted before analysis using high performance anion exchange chromatography in combination with pulsed amperometric detection (HPAEC‐PAD). The oligosaccharide peaks were annotated using dextranase‐treated IMMP‐94 as a standard. The dextranase‐treated IMMP‐94 was prepared as follows: 0.25 unit of dextranase from *Chaetomium erraticum* (Sigma‐Aldrich, St. Louis, MO, USA) was added to 5 mg IMMP‐94 in 1 mL of 0.1 M sodium maleate buffer containing 5 mM CaCl_2_ at pH 6, and incubated at 37 °C for 30 min. The reaction was stopped by heating at 99 °C for 5 min, and the supernatant was diluted five times for HPAEC analysis after centrifuging at 18 600 × *g* for 10 min at room temperature.

Ten μL of sample was injected to a Dionex ICS 5000 system (Dionex) with a CarboPac PA‐1 column (250 mm × 2 mm ID) and a CarboPac PA guard column (25 mm × 2 mm ID). The column temperature was 20 °C. The flow rate of the two mobile phases (A) 0.1 M NaOH and (B) 1 M NaOAc in 0.1 M NaOH was set to 0.3 mL min^−1^. The gradient elution was applied as follows: 0–40 min, 0–40% B; 40–40.1 min, 40–100% B; 40.1–45 min, 100% B; 45–45.1 min, 100–0% B; 45.1–60 min, 0% B. The elution was monitored by a PAD (Dionex ISC‐5000 ED). Chromeleon 7.1 software (Dionex) was used to process data from HPAEC.

#### 2.3.3 Extraction and Activity of Bacterial Enzymes

Part of the fermentation digest (0.4 mL) was snap frozen in liquid nitrogen and stored at −80 °C before enzyme extraction. The following fermentation digests were selected according to HPAEC results (see Section 3 for further details): IMMP blank, IMMP‐27 at 12 and 48 h, IMMP‐dig27 at 12 and 24 h, and IMMP‐94 at 12 and 24 h. Protein extraction was performed as described elsewhere^14^ with some modifications. To obtain the fraction of extracellular enzymes (EE), the fermentation digest was first centrifuged (21 000 × *g*, 4 °C, 10 min), and the supernatant was applied on a 10 kDa centrifugal filter (VWR, Amsterdam, The Netherlands) at 4 °C and 18 600 × *g* to remove any mono‐ and oligosaccharides produced during fermentation. A volume of 0.4 mL 25 mM MES buffer pH 5.8 containing 1 mM phenylmethylsulfonyl fluoride and 1 mM dithiothreitol was used to reconstitute the retentate (EE). The pellet from the first centrifugation step was washed once with 1.5 mL buffer, centrifuged again and then suspended in 0.4 mL of the same MES buffer. The suspension was sonicated at 30% amplitude for 30 s and repeated three times with 40 s break in between.^13^ The supernatant after centrifugation was used as cell‐associated enzymes (CE).

The enzyme activity of EE and CE toward PNP‐glucose substrates and starch was determined using a color reaction, as described previously^14^ with some modifications. In the glycosidase assay, only PNP‐α‐d‐glucopyranoside and PNP‐β‐d‐glucopyranoside were included as substrates. In the polysaccharide assay, soluble potato starch (Sigma‐Aldrich) and IMMP‐94 were used as substrates. Potato starch was incubated at 99 °C until solubilized. The substrate (3.125 mg mL^−1^) was mixed with enzyme extracts in a 4:1 ratio, yielding a final substrate concentration of 2.5 mg mL^−1^. The amount of reducing sugar released after 1 h incubation was determined by 4‐hydroxybenzoic acid hydrazide (PAHBAH) assay using glucose as a standard.^13^ Enzyme activities were expressed in mU (nmol‐reduced‐end‐formed* mL‐digest^−1^ min^−1^).

#### 2.3.4 Analysis of SCFAs and Other Organic Acids by GC‐FID and HPLC‐RI

Determination of SCFAs (acetic acid, propionic acid, and butyric acid) by gas chromatography (GC) and of lactic acid and succinic acid by high performance liquid chromatography (HPLC) was done as described previously^22^ with some modifications. For GC, 70 μL of twofold diluted supernatant of the fermentation digest was mixed with 70 μL 0.15 M oxalic acid and allowed to stand at room temperature for 30 min. Then 199 μL water and 1 μL of 5 mg mL^−1^ 2‐ethylbutyric acid were added. The temperature profile during GC analysis was as follows: from 100 to 165 °C at 5 °C min^−1^, then held at 165 °C for 1 min. Chromeleon 7.1 software (Dionex) was used to process data from HPLC. Xcalibur software (Thermo Scientific, Breda, The Netherlands) was used to process data from GC.

#### 2.3.5 DNA Extraction, 16S Ribosomal RNA Gene Sequencing, and Microbial Composition Analysis

The pellets obtained from the centrifugation of fermentation digest were snap frozen in liquid nitrogen, stored at −80 °C and used for microbial composition analysis. Total bacterial DNA was extracted using the Maxwell 16 Total RNA system (Promega, Wisconsin, USA) with Stool Transport and Recovery Buffer (STAR; Roche Diagnostics Corporation, Indianapolis, IN). Briefly, bacterial pellets were homogenized with 0.25 g of sterilized 0.1 mm zirconia beads and three glass beads (2.5 mm) in 300 μL STAR buffer for 3 × 1 min at 5.5 m s^−1^ using a bead beater (Precellys 24, Bertin Technologies), with cooling on ice for 1 min in between. Samples were incubated with shaking at 100 rpm for 15 min at 95 °C and pelleted by 5 min centrifugation at 4 °C and 14 000 × *g*. Supernatant was removed and the pellets were processed again using 200 μL fresh STAR buffer. Samples were incubated at 95 °C and centrifuged as before. Supernatant was removed, pooled with the first supernatant and 250 μL was used for purification with Maxwell 16 Tissue LEV Total RNA Purification Kit (AS1220) customized for DNA extraction in combination with the STAR buffer. DNA was eluted with 50 μL of DNase‐ and RNase‐free water (Qiagen, Hilden, Germany). DNA concentrations were measured with a NanoDrop ND‐1000 spectrophotometer (NanoDrop Technologies, Wilmington, DE, USA) and adjusted to 20 ng μL^−1^ with DNase‐ and RNase‐free water. The V4 region of 16S ribosomal RNA (rRNA) genes was amplified. PCR reactions were done in duplicates, each in a total volume of 50 μL and containing 20 ng of template DNA. Each sample was amplified with a unique barcoded primer pair 515F‐n (5′‐GTGCCAGCMGCCGCGGTAA‐) and 806R‐n (5′‐RGGATTAGATACCC) (10 μM each per reaction^23^), 1× HF buffer (Finnzymes, Vantaa, Finland), 1 μL dNTP Mix (10 mM each; Roche Diagnostics GmbH, Mannheim, Germany), 1 U Phusion Hot Start II High Fidelity DNA Polymerase (Finnzymes, Vantaa, Finland), and 36.5 μL of DNase‐ and RNase‐free water. The amplification program included 30 s initial denaturation step at 98 °C, following by 25 cycles of denaturation at 98 °C for 10 s, annealing at 56 °C for 10 s, elongation at 72 °C for 10 s, and a final extension at 72 °C for 7 min. The PCR product presence and size (≈290 bp) was confirmed with gel electrophoresis using the Lonza FlashGel System (Lonza, Cologne, Germany). Seventy unique barcode tags used in each library and artificial control (mock) communities representative of human intestinal microbiota were included.^23^ PCR products were purified with HighPrep PCR kit (MagBio Genomics, Alphen aan den Rijn, The Netherlands), and DNA concentrations were measured with Qubit dsDNA BR Assay Kit (Life Technologies, Leusden, The Netherlands). Hundred nanograms of each barcoded sample was added to an amplicon pool that was subsequently concentrated with HighPrep PCR kit to 20 μL volume. The concentration was measured with Qubit dsDNA BR Assay Kit and adjusted to 100 ng μL^−1^ final concentration. The libraries were sent for adapter ligation and HiSeq sequencing (GATC‐Biotech, Konstanz, Germany). Data processing and analysis was carried out using NG‐Tax.^23^ Diversity analyses were carried out in QIIME.^24^, ^25^ Relative abundance at genus level was used for calculating pairwise Pearson correlation scores between biological replicates, and the values for the different taxa were averaged for each replicate pair.

## 3 Results and Discussion

### 3.1 Fermentation Reproducibility

The in vitro fermentation experiments were run in two separate batches, the first one using IMMP‐27 and IMMP‐94, and the second batch with IMMP‐dig27 and IMMP‐96. Between‐batch similarity was estimated based on Pearson correlation scores of genus‐level microbiota composition data for the IMMP blank samples from different batches, at times 0, 24, and 48 h and were 0.98, 0.94, and 0.88, respectively. A high reproducibility for the results between the two batches was found, validating the between‐batch comparisons to be carried out when necessary. Pearson correlation scores also showed high levels of similarity between the biological duplicates at genus level (average Pearson score of 0.97, SD ± 0.03 for IMMP treatment groups and 0.90, SD ± 0.22 for IMMP blank groups).

### 3.2 Physicochemical Characterization of IMMP‐94 and IMMP‐96

**Figure 2:**
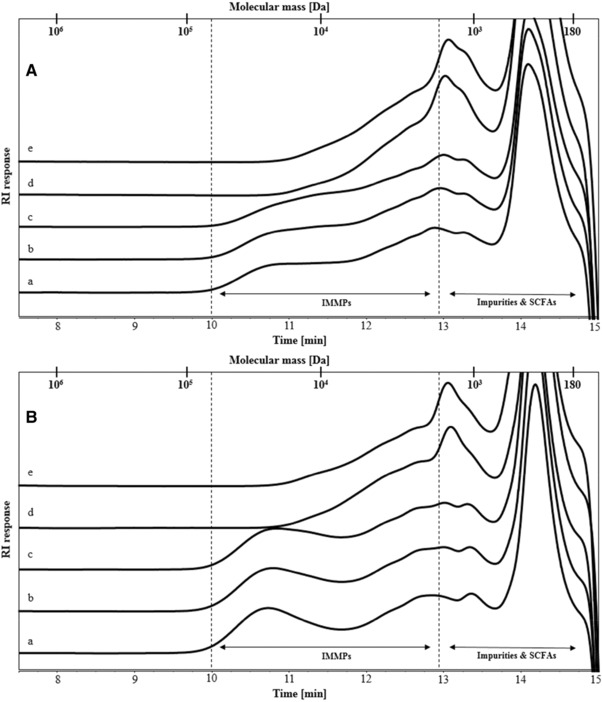
High performance size exclusion chromatography (HPSEC) elution patterns of A) IMMP‐94 originating from potato starch, and B) IMMP‐96 originating from Etenia, a) before and after in vitro fermentation for b) 6 h, c) 12 h, d) 24 h, and e) 48 h. Calibration of the system with pullulan standards is indicated.

IMMP‐94 and IMMP‐96 contained high percentages of α‐1,6‐linkages as a result of including the debranching enzyme pullulanase during the synthesis by GTFB. The two IMMPs were derived from different starches, namely normal potato starch and amylomaltase‐treated potato starch. Amylomaltase treatment results in a disappearance of the amylose fraction and a broader chain length distribution of the amylopectin fraction, due to the disproportionation effect of the amylomaltase enzyme.^26^ In order to verify potential differences in the molecular weight distribution, both IMMPs were compared by HPSEC using samples prior to fermentation (**Figure** 2A,B, lines a). IMMP‐94 showed a broad molecular weight distribution with populations being eluted between 10 and 12.9 min (1.7–65 kDa). IMMP‐96 showed a slightly more clear bimodal distribution, with higher RI response toward both ends of the same elution window (10–12.9 min), indicating that IMMP‐96 contained both shorter and longer chains and fewer medium length chains, as compared to IMMP‐94.

### 3.3 Influence of IMMP Molecular Weight Distribution on Its in Vitro Fermentation

#### 3.3.1 Polymer Degradation and Oligosaccharide Formation upon in Vitro Fermentation of IMMP‐94 and IMMP‐96

**Figure 3:**
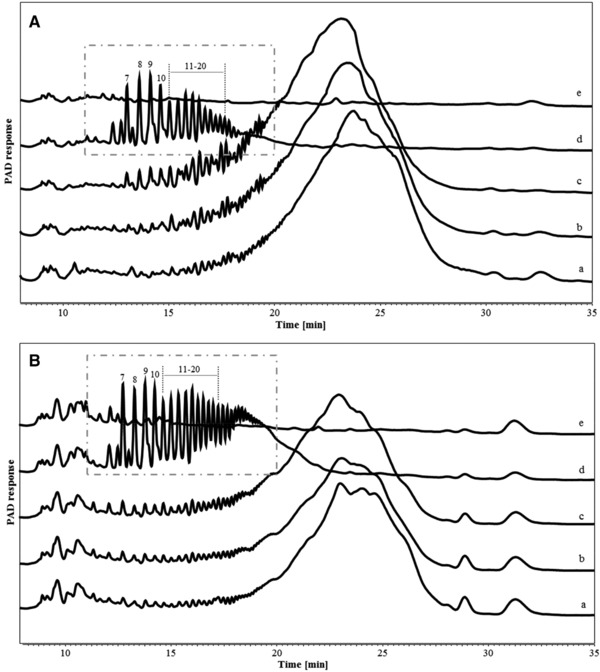
High performance anion exchange chromatography (HPAEC) elution patterns of A) IMMP‐94 originating from potato starch, and B) IMMP‐96 originating from Etenia 457, a) before and after in vitro fermentation for b) 6 h, c) 12 h, d) 24 h, and e) 48 h. Isomalto‐oligosaccharides are annotated in a box, with the number indicating their degree of polymerization (DP).

The degradation of IMMPs during in vitro fermentation was monitored by HPSEC up to 48 h (Figure 2). The fermentation behavior of IMMP‐94 and IMMP‐96 was similar, and the same types of oligomeric dextran fragments were formed and utilized in time. For both IMMPs, the HPSEC elution patterns remained the same during the first 12 h, followed by a shift in molecular size from larger to smaller molecules from 12 to 24 h. No further difference was noted between 24 and 48 h of incubation suggesting that the degradation of the polysaccharides fraction of IMMP‐94 and IMMP‐96 mainly took place between 12 and 24 h of fermentation. To have a better overview of smaller size molecules being formed during fermentation, HPAEC was performed (**Figure** 3). For both IMMPs, a broad peak being eluted between 20 and 25 min was seen during the first 12 h of fermentation. This peak included a wide range of not well‐separated IMMP molecules, which partly corresponded to the 10–65 kDa population in the HPSEC chromatograms (Figure 2A,B, lines a, b, c). At 24 h of fermentation, these polymers had disappeared, and a series of well‐separated oligosaccharide peaks which eluted between 11 and 20 min could be observed. The oligosaccharide peaks were annotated according to the HPAEC elution pattern of IMMP‐94 treated with a pure dextranase from *C. erraticum* (results not shown). The oligosaccharide fraction of the fermentation digest comprised α‐(1→6) linked IMOs with a DP of 7 to over 20. IMOs with DP <7 were absent at 24 h, which could be due to instant consumption of smaller oligosaccharides by the microbiota during fermentation, indicating a preference of the microbiota for the utilization of small molecules. At 48 h of incubation, the oligosaccharide fraction had disappeared, and no carbohydrate peaks were present in the chromatogram (Figure 3). Overall, the HPAEC results of IMMP‐94 and IMMP‐96 were in accordance with HPSEC results.

Despite similarities in the fermentation behavior of IMMP‐94 and IMMP‐96, the overall rate of fermentation of IMMP‐96 was slower (Figure 3), as indicated by the presence of polymeric material being eluted between 18 and 20 min after 24 h of incubation. This difference in fermentation rate could be due to the difference in chain length distributions of the two IMMPs. This finding agreed with a previous research which reported that IMOs of different chain length led to different utilization and fermentation rate when using human fecal microbiota.^27^

#### 3.3.2 pH and Production of Organic Acids upon in Vitro Fermentation of IMMP‐94 and IMMP‐96

**Figure 4:**
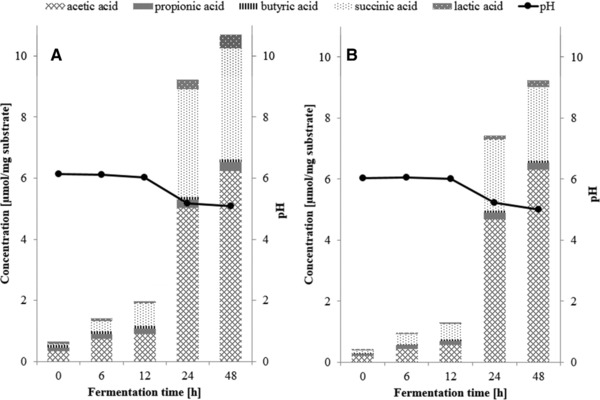
Concentrations of short chain fatty acids, lactic acid, and succinic acid and the pH (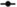) during in vitro fermentation with human fecal inoculum of A) IMMP‐94 and B) IMMP‐96.

Analysis of the pH of fermentation digesta and organic acid production at different time points confirmed that the fermentation of IMMP‐94 and IMMP‐96 started after 12 h of incubation (**Figure** 4). For both IMMP‐94 and IMMP‐96, the pH remained stable at around pH 6.2 during the first 12 h, followed by a decrease to around pH 5.2 at 24 h, and a slight further decrease at 48 h (Figure 4). It is noteworthy that the drop of the pH to 5.0 at 48 h was larger as compared to a drop of pH to 6.0 at 48 h previously observed for resistant gluco‐dextrin fermentation in a comparable setup.^14^ The pH decreased as a result of organic acid production. In line with the change of pH, the largest increase in the concentration of SCFAs was observed from 12 to 24 h, followed by a further increase from 24 to 48 h (Figure 4). Acetic acid, propionic acid, and butyric acid are in general the three main SCFAs produced during in vitro fermentation of carbohydrates. Lactic acid and succinic acid should also be taken into consideration, since they are intermediates in SCFA production during fermentation.^28^

For both IMMPs, the most predominant SCFA produced was acetic acid, with minor amounts of propionic acid and butyric acid (Figure 4). Unexpectedly, the second most produced organic acid for both IMMPs was succinic acid. Succinic acid is an intermediate of intestinal SCFA production, and is utilized by members of the phylum Bacteroidetes and the family Veillonellaceae to form propionic acid.^29^, ^30^, ^31^ In the current study, however, succinic acid accumulated during the incubation without further conversion. This accumulation of succinic acid can also explain the lower pH at the end of fermentation, since succinic acid has a p*K_a1_* of 4.2,^32^ which is lower than that of other SCFAs (approximately 4.8).^4^

The drop of pH and the predominant production of acetic acid are in line with a previous report on in vitro fermentation of IMMPs.^6^ Formation of succinic acid was not reported in that study; however, it should be noted that only acetic acid and propionic acid were measured. Information about succinic acid was also not presented for studies where IMOs or dextrans were fermented,^8^, ^10^, ^11^ but was reported for fermentation studies where other prebiotics were used as substrate, for example, lactulose and inulin.^33^, ^34^, ^35^ It has been reported previously that *Bacteroides fragilis* produced acetate and succinate mainly in the presence of sufficient carbon source, whereas it converted succinate to propionate when carbon sources were limited.^28^ An in vivo study was performed in collaboration with the University Medical Centre Groningen (The Netherlands), where IMMPs were fed to mice (unpublished results). Also in the mice feces, significant amounts of succinic acid were found, providing additional evidence that the production and accumulation of succinic acid during IMMPs’ fermentation was not an artifact of the in vitro fermentation setup.

The degradation of IMMPs started later and continued over a longer time than that of other commonly studied prebiotics. In the comparable in vitro fermentation setup, utilization of FOS started at around 2 h after fecal inoculation and was completed within 9 h.^36^ Therefore, IMMPs can be considered to be a slowly fermentable fiber, although a direct comparison between the substrates might be necessary to unequivocally confirm observations described here. Slowly fermentable fibers are of great interest, because most colonic diseases occur distally, where proteolytic fermentation may take place when carbohydrates are lacking.^2^, ^37^ The slow fermentability of IMMPs makes them beneficial to gut health by increasing the delivery of SCFAs to the distal colon. Besides, given the fact that IMOs of DP <10 were shown to be bifidogenic,^9^, ^38^, ^39^ results presented here indicate that IMMPs are a good fiber source to make these IMOs available for the fermentation by the colonic microbiota.

### 3.4 Physicochemical Characterization of IMMP‐27 and IMMP‐dig27

Starch, due to its high content of α‐(1→4) linked glucosyl residues, is mostly digested in the human small intestine. In contrast, when mixed with α‐(1→6) linked glucosyl moieties such as in IMMPs, it is possible that part of the α‐(1→4) linked glucoses could escape digestion and enter the large intestine. To investigate the influence of α‐(1→4) linked glucosyl residues on the fermentation of α‐(1→6) linked glucose segments by colonic microbiota, the in vitro fermentation of IMMP‐27 and IMMP‐dig27 were compared. IMMP‐27 contains 27% α‐(1→6) linked glucosyl residues, whereas IMMP‐dig27 is the α‐(1→6) glucan enriched fraction of IMMP‐27, after being treated with an excess of α‐amylase and amyloglucosidase that removed >70% of glucose moieties.

**Figure 5:**
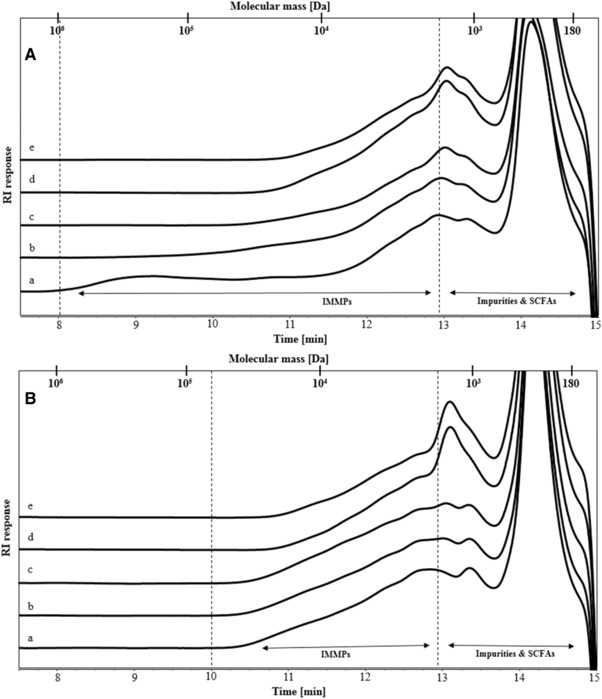
High performance size exclusion chromatography (HPSEC) elution patterns of A) IMMP‐27 and B) IMMP‐dig27, a) before and after in vitro fermentation for b) 6 h, c) 12 h, d) 24 h, and e) 48 h. Calibration of the system with pullulan standards is indicated.

The molecular size distribution of IMMP‐27 and IMMP‐dig27 was determined by HPSEC (**Figure** 5A,B, lines a). The overall molecular size of IMMP‐27 was larger than that of IMMP‐dig27. Molecules that eluted at 8–10 min (65–850 kDa) in IMMP‐27 were not observed in IMMP‐dig27, indicating that this fraction of molecules was digested to smaller fragments due to the removal of α‐(1→4) linked glucose moieties by α‐amylase and amyloglucosidase.

### 3.5 Influence of α‐1,4‐Linkages on Bacterial Utilization of α‐1,6‐Linked Glucose during in Vitro Fermentation of IMMPs

#### 3.5.1 Polymer Degradation and Oligosaccharide Formation upon Fermentation of IMMP‐27 and IMMP‐dig27

**Figure 6:**
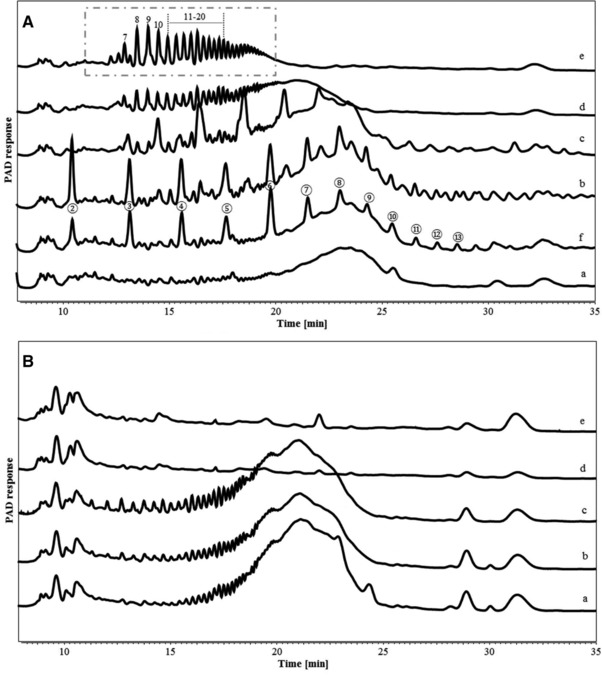
High performance anion exchange chromatography (HPAEC) elution patterns of A) IMMP‐27 and B) IMMP‐dig27, a) before and after in vitro fermentation for f) 15 min, b) 6 h, c) 12 h, d) 24 h, and e) 48 h. Isomalto‐oligosaccharide peaks (7–20) in a box and maltodextrin peaks (②–⑬) are annotated, with the number indicating the DP.

The change in molecular size distribution of IMMP‐27 and IMMP‐dig27 during in vitro fermentation was monitored using HPSEC (Figure 5). For IMMP‐27, HPSEC chromatograms showed differences between 0 and 6 h, with molecules ranging in size between 65 and 850 kDa being degraded within 6 h of fermentation. After 6 h, the chromatograms of IMMP‐27 did not show any further increase in the proportion of the smaller molecules which eluted at 8–10 min (65–850 kDa). In contrast, the chromatograms of IMMP‐dig27 remained the same in the first 12 h, and there was a shift in the molecular size distribution to smaller molecules between 12 and 24 h. No changes in the elution patterns were observed from 24 to 48 h, indicating that the degradation of IMMP polymers was completed. The oligomer profiles of IMMP‐27 and IMMP‐dig27 during fermentation obtained by HPAEC showed that for IMMP‐27, α‐1,4‐linked maltodextrin peaks were already present at 15 min, and were still present at 6 h (**Figure** 6). At 12 h, these maltodextrin peaks were hardly present, whereas new peaks, probably representing oligosaccharides consisting of both α‐(1→4) and α‐(1→6) linkages, became more apparent (Figure 6). At 24 h, a series of well‐separated α‐1‐6‐linked IMO peaks which eluted between 11 and 20 min appeared, and a broad fraction eluting between 20 and 24 min representing unseparated dextran oligomers of higher DPs was clearly seen. The peaks of IMOs (11–20 min) were still present at 48 h of fermentation, whereas the unseparated fraction (20–24 min) disappeared. For IMMP‐dig27, with hardly any α‐1,4‐linkages present in the substrate, the IMMP molecules remained intact during the first 12 h of fermentation. However, no carbohydrates were detected at 24 h of fermentation, indicating that a very quick and complete fermentation took place between 12 and 24 h.

The different degradation patterns of IMMP‐27 and IMMP‐dig27 suggest that in the in vitro fermentation model, human fecal microbiota could utilize the α‐(1→4) linkages directly, whereas α‐(1→6) linkages were utilized only after the α‐(1→4) linkages were depleted. Different enzymes are required to digest α‐(1→4,6) linkages, and bacteria present in the fecal inoculum could be induced to produce corresponding hydrolytic enzymes by the presence of specific substrates in the colon.^40^ However, when mixtures of compounds are present, the availability of one substrate could delay the fermentation of another, possibly less favorable substrate.

Our results suggest that the presence of α‐(1→4) linked glucosyl residues could postpone the utilization of α‐(1→6) linked glucosyl residues in vitro and that fermentation of IMMPs with high levels of α‐(1→6) linkages may require colonic microbiota to undergo an adaptation period. Furthermore, this adaptation period might relate to the molecular size of the α‐(1→6) glucan chains. The fermentation behavior of IMMP‐dig27 resembled that of IMMP‐94 and IMMP‐96, in line with the facts that all three substrates are rich in α‐(1→6) linked and depleted in α‐(1→4) linked glucose residues.

The complete degradation of IMMP‐dig27, however, was faster than that of the other two IMMPs. This could be explained by the smaller molecular sizes of IMMP‐dig27 “dextran” segments as compared to IMMP‐94 and IMMP‐96, indicating that the fermentation of α‐(1→6) linkages is quicker for smaller IMMP molecules. Therefore, the fermentation of IMMPs depends not only on the presence of α‐(1→4) linkages, but also on the molecular length distribution of IMMPs, although it would be necessary to further investigate whether α‐(1→4) linked glucosyl residues still present within the IMMPs would escape digestion and enter the colon in vivo.

#### 3.5.2 pH and Production of Organic Acids upon Fermentation of IMMP‐27 and IMMP‐dig27

**Figure 7:**
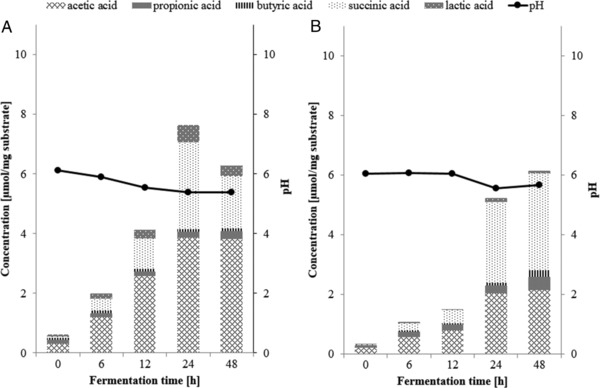
Concentrations of short chain fatty acids, lactic acid, and succinic acid present and the pH (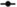) during in vitro fermentation with human fecal inoculum of A) IMMP‐27 and B) IMMP‐dig27.

For IMMP‐27, the pH dropped continuously from the beginning of the fermentation until 24 h, which agrees with the steadily increasing level of SCFAs, lactic acid, and succinic acid produced during the first 24 h (**Figure** 7). From 24 to 48 h, the pH remained stable and the concentrations of lactic acid and succinic acid decreased. During the fermentation of IMMP‐dig27, the pH dropped between 12 and 24 h, concomitant with the most pronounced increase in the level of total organic acids, resembling the results for IMMP‐94 and IMMP‐96. The pH profiles and SCFAs production of IMMP‐27 and IMMP‐dig27 fermentation further confirmed that the human fecal microbiota used here readily utilized the α‐(1→4) glucan chains, whereas the utilization of the α‐(1→6) glucan chains was delayed. A slight increase of pH (from 5.5 to 5.7) was observed between 24 and 48 h when fermenting IMMP‐dig27. This could be an indication of proteolytic fermentation, of which one of the end‐products is ammonia (not measured in this study).^41^ The onset of proteolytic fermentation was possibly a result of carbohydrate depletion of IMMP‐dig27 after 24 h fermentation.

Furthermore, acetic acid and succinic acid were the two major products for IMMP‐27 and IMMP‐dig27, as reported above for IMMP‐94 and IMMP‐96. Overall, the production of SCFAs with IMMP‐dig27 resembled that with IMMP‐94 and IMMP‐96, except that the production of acetic acid was much lower in the final concentration for IMMP‐dig27 between 24 and 48 h (Figures 4 and 7). The lower production of acetic acid explained the slightly higher pH at 48 h in the fermentation of IMMP‐dig27 (5.7) compared to that of IMMP‐94 (5.0) and IMMP‐96 (5.0). Furthermore, within the first 12 h of fermentation of IMMP‐27, where mainly the α‐(1→4) linked glucoses were utilized by fecal bacteria, succinic acid was already produced in large quantity (Figure 7). Therefore, the production of succinic acid was not specific to fermentation of the α‐(1→6) linked glucosyl residues of IMMPs.

### 3.6 Enzyme Activity upon Fermentation of IMMPs

During in vitro fermentation, IMMP molecules with higher DP were degraded into molecules with lower DP by enzymes that were produced by fecal microbiota, followed by further degradation into glucose, which was then utilized by the bacteria present. To investigate which enzymes were produced during IMMP fermentation, proteins were extracted from fermentation digests at selected time points, chosen based on the HPAEC patterns: IMMP‐94 (12 and 24 h), IMMP‐27 (12 and 48 h), and IMMP‐dig27 (12 and 24 h). These time points indicated the time before the α‐(1→6) glucan chains started to be degraded (all three IMMPs), the time when IMOs of DP 7–20 were predominantly present (IMMP‐27 and IMMP‐94) or even fully utilized (IMMP‐dig27). IMMP‐94 was used to represent IMMPs that were rich in α‐(1→6) linkages. Besides, the IMMP blank which contained inoculum with no IMMPs at time 0 h was included as the baseline of enzyme activity. From all time points, two types of enzyme extracts were obtained: extracellular enzyme extract and cell‐associated enzyme extract. Four substrates, PNP‐α‐d‐glucopyranoside, PNP‐β‐d‐glucopyranoside, potato starch, and IMMP‐94, were tested to determine the presence and activity of α‐ and β‐1,4‐glucosidases, starch‐degrading enzymes, and dextran‐degrading enzymes (**Table** 1).

**Table 1:** Enzyme activity in mU (nmol mL‐digest^−1^ min^−1^) of enzyme extracts from in vitro fermentation samples. EE, extracellular enzymes; CE, cell‐associated enzymes

			Enzyme assay substrates			
In vitro fermentation substrates	Fermentation time [h]	Enzyme extract	PNP‐α‐d‐glucopyranoside	PNP‐β‐d‐glucopyranoside	Soluble potato starch	IMMP‐94
Inoculum blank	0	EE	31	27	–	–
		CE	0.5^a^	0.2	–	–
IMMP‐94	12	EE	32	47	43	46
		CE	71b)	2.5	1	0.4
	24	EE	7b)	2	20	69
		CE	448	46	6	29
IMMP‐27	12	EE	45	25	14	21
		CE	127b)	10^a^	1	–
	48	EE	61	4	60	20
		CE	101^a^	13	28	8
IMMP‐dig27	12	EE	64^a^	N.A.	36	31
		CE	1^a^	0.03	1	7
	24	EE	25	6	17	32
		CE	76	7	4	74

a Results given by single test; ^b)^results given by duplicates; all other results given by triplicates. –, not detectable; N.A., not analyzed.

At baseline (IMMP blank, 0 h), all enzyme activities measured were neglectable, especially the starch/dextran‐degrading enzymes, as neither CE nor EE showed detectable activity toward soluble potato starch or IMMP‐94 (Table 1). When IMMPs were present during the fermentation, the enzyme activities toward PNP‐α‐d‐glucopyranoside increased at 12 h, especially in CE of IMMP‐27, 12 h (127 mU compared to 0.5 mU in IMMP blank). For both IMMP‐94 and IMMP‐dig27, the enzyme activities of CE toward PNP‐α‐d‐glucopyranoside at 24 h were much higher than those at 12 h. In general, the enzyme activity toward PNP‐α‐d‐glucopyranoside was much higher than the activity toward PNP‐β‐d‐glucopyranoside for all enzyme extracts, suggesting that the microbiota was induced to produce enzymes to degrade the α‐glucans used in this study. Enzyme activities toward soluble potato starch and IMMP‐94 were also higher when IMMPs were present as substrates in the fermentation, and the enzyme activities of EE were much higher than that of CE. The EE enzyme extracts of IMMP‐94 showed an increasing activity toward IMMP‐94 from 12 h (46 mU) to 24 h (69 mU), whereas a declining activity toward soluble potato starch from 12 h (43 mU) to 24 h (20 mU) was observed. This confirms that the production of α‐(1→6) linkage hydrolytic enzymes was induced by the presence of α‐(1→6) linkage‐rich substrates after the disappearance of α‐(1→4) linkages. The decrease in activity of α‐(1→4) linked glucose endo‐acting enzyme was most probably due to the absence of starch, and the α‐(1→4) linked glucose hydrolytic enzyme that was found active at the beginning was no longer produced during the later stages of the fermentation.

The overall distribution of the four enzyme activities in CE and EE followed a certain tendency: activities toward soluble potato starch and IMMP‐94, that is, α‐amylase and dextranase, were higher in EE than in CE, whereas activities toward PNP‐α/β‐d‐glucopyranoside were higher in CE than in EE. This suggests that α‐(1→4) and α‐(1→6) linked glucose polysaccharide‐degrading enzymes, which comprise mainly of endo‐acting enzymes,^14^ were excreted by microbes to cleave IMMP polysaccharides into smaller oligosaccharides. These smaller oligosaccharides could then be taken up by microbial cells to be further degraded by glucosidases, which are exo‐acting enzymes. This agrees with previous findings that exo‐acting enzymes were mostly cell‐bound whereas end‐acting enzymes were mostly extracellular.^14^ Also the absence of IMOs of DP lower than seven in the well‐separated IMO fraction in the HPAEC chromatograms (Figures 3 and 6) seems to match this theory, because bacterial cells, together with the smaller oligosaccharides that had already passed the cell membrane, were removed from fermentation digest by centrifugation before HPAEC analysis.

According to HPAEC (Figure 6), degradation of IMMP‐27 was mainly targeting α‐(1→4) linkages in the first 12 h, and switched to α‐(1→6) linked glucosyl residues afterward. Furthermore, at 48 h, IMOs of DP 7–20, which were products of degradation of IMMP polysaccharides by endo‐acting enzymes, were present. This means that the α‐(1→4) linkage‐degrading enzymes were active during the first 12 h of fermentation, whereas afterward, glycanase activity was taken over by the α‐(1→6) linkage‐degrading enzymes. However, this did not agree with the enzyme activities measured during fermentation of IMMP‐27: the combined CE and EE enzyme activities toward soluble potato starch were higher at 48 h (88 mU) than at 12 h (15 mU). In addition, the enzyme extracts of IMMP‐27‐48 h showed higher combined CE and EE enzyme activities toward soluble potato starch (88 mU) than toward IMMP‐94 (28 mU). This suggests that the production of α‐1,4‐linkage‐degrading enzymes was not suppressed after the substrates were depleted.

As to IMMP‐dig27, the combined CE and EE enzyme activity toward soluble potato starch declined from 12 h (37 mU) to 24 h (21 mU), whereas the activity toward IMMP‐94 increased from 12 h (38 mU) to 24 h (106 mU). This observation suggested that the microbial enzyme production of IMMP‐dig27 fermentation resembled that of IMMP‐94 fermentation. This agreed with the results of molecular degradation patterns and SCFA production, as discussed above.

### 3.7 Microbiota Composition during Fermentation of IMMPs

**Figure 8:**
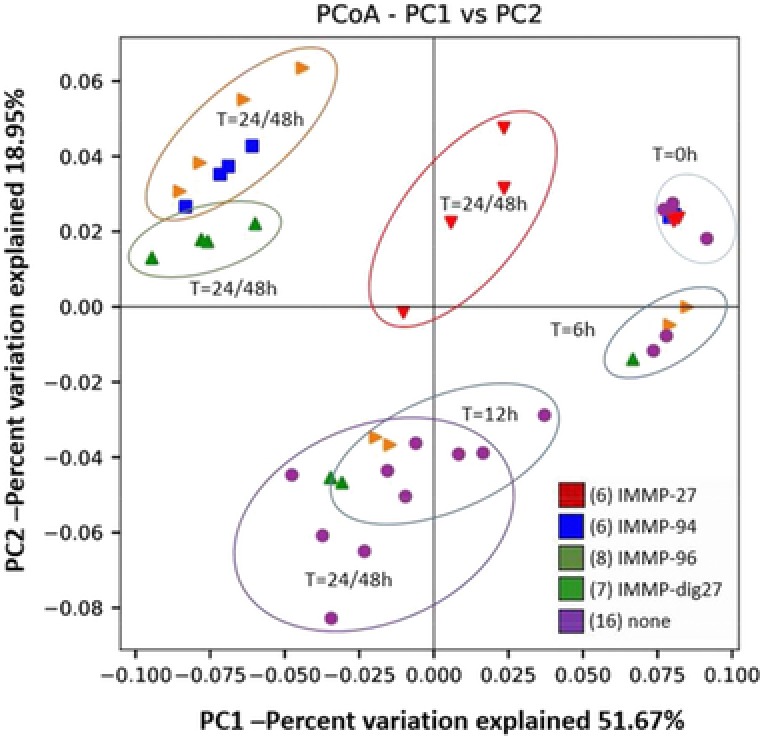
PCoA based on weighted (relative abundance) UniFrac distances between observed microbial communities for in vitro fermentation of IMMPs with human fecal inoculum at different time points.

**Figure 9:**
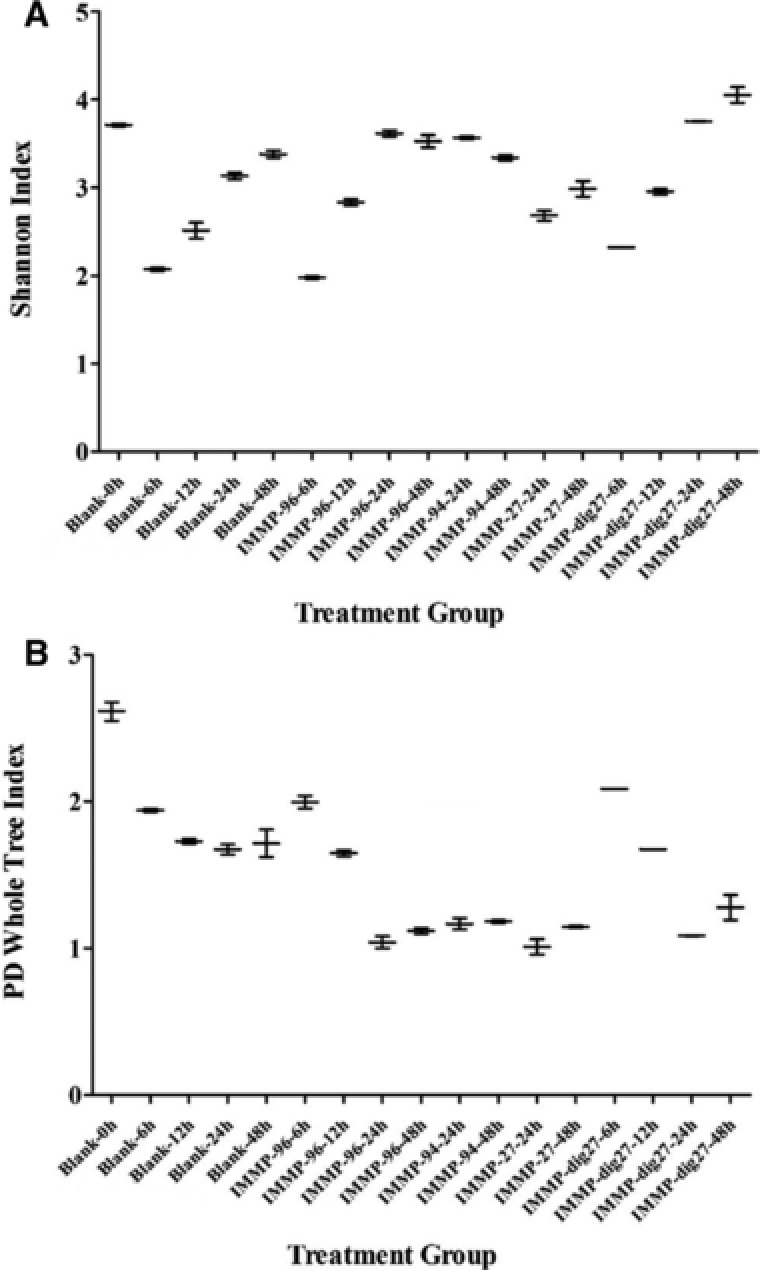
Microbial alpha‐diversity estimates, including A) Shannon diversity index, B) Phylogenetic Diversity Whole Tree for in vitro fermentation of IMMPs with human fecal inoculum at different time points.

**Figure 10:**
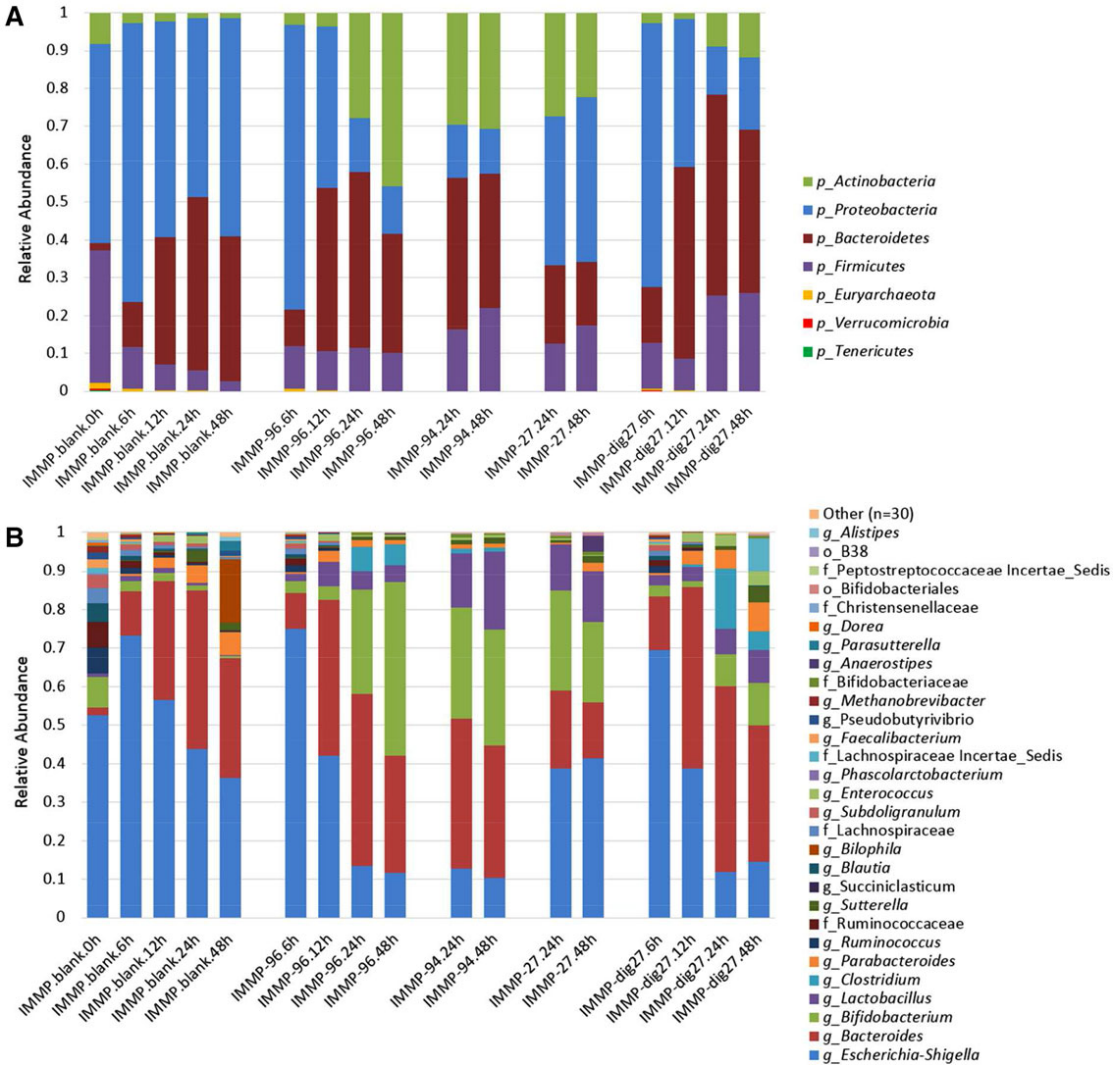
Relative abundance of taxa detected during in vitro fermentation of IMMPs with human fecal inoculum at different time points, considering A) phylum and B) genus levels.

The microbiota composition during the fermentation of IMMPs was analyzed to evaluate the prebiotic potential of IMMPs, and to make a link with the structural changes of IMMPs and production of SCFAs, lactic acid, and succinic acid. Multivariate analysis of bacterial community dynamics over time in the different in vitro fermentations, using weighted Unifrac distances as a measure for differences in microbial composition, showed a directional shift in community composition in relation to incubation time and the type of IMMPs used. A strong segregation of samples with IMMPs present after 24/48 h of incubation can be seen in **Figure** 8. This indicates that both the duration of incubation and the presence of different IMMP substrates played an important role in shaping the microbial communities in vitro. A similar segregation of samples was also found with unweighted analyses that only take the presence and the absence of microbial groups into account (data not shown). The microbial alpha diversity, as determined based on Shannon's diversity index, changed as the fermentation progressed and decreased in the blank, but increased in digesta with the IMMPs present (**Figure** 9). Shannon's diversity index accounts for both abundance and evenness of the species present. There was a high predominance of *Escherichia–Shigella* group at the beginning of the fermentation, possibly due to the presence of residual amounts of oxygen during initial inoculum activation. As fermentation progressed, the presence of IMMPs and the depletion of oxygen enabled growth of other bacterial groups leading to an increase in the evenness of the community. Although the microbiota composition at the start of the fermentation was different from that normally found in feces of healthy adults, it is interesting to note that such a dysbiotic community was “normalized” by IMMPs toward a more typical colonic microbiota. It is tempting to speculate that this “normalization” effect might also occur in vivo and could facilitate ecosystem recovery following situations of dysbiosis (e.g., after diarrhea). In the IMMP blank sample, the ecosystem was starved, thus the growth of other bacteria groups was much slower. Phylogenetically weighted species richness, as measured by the Phylogenetic Diversity (PD) Whole Tree index, decreased in all treatment groups in the first hours of incubation, whereas it remained relatively stable after 24 h. Despite high structural similarities between IMMP‐94 and IMMP‐96, Pearson correlation scores at genus level were 0.77, 0.89, and 0.34 at times 0, 24, and 48 h, respectively, suggesting different microbial response patterns toward these two substrates. In line with this observation, the average relative abundance of different phyla changed with time, and was influenced by the type of IMMP being fermented (**Figure** 10A). Levels of Proteobacteria decreased in all groups until 24 h of incubation and remained stable or slightly increased at 48 h. This was accompanied by a gradual increase in Bacteroidetes up to 24 h, followed by decrease at 48 h. Firmicutes showed a rapid decrease in abundance at 6 h and gradual increase at later time points, except for IMMP blank where their relative abundance continued to decline. The levels of Actinobacteria were very low, and decreased to 4.7% in the IMMP blank at 48 h. Their relative abundance was higher in the IMMP digesta as compared to the IMMP blank.

At the genus level, four bacterial taxa, namely *Escherichia–Shigella, Bacteroides, Bifidobacterium*, *and Lactobacillus* were predominant in all IMMP digesta, with their combined relative abundance ranging from 41% to 97% of all detected reads (Figure 10B). The detailed relative abundance of taxa during IMMPs’ fermentation at genus level is given in Table S1, Supporting Information. The duration of in vitro fermentation was positively correlated with the increase of *Bacteroides* and a corresponding decrease in *Escherichia–Shigella*. The presence of IMMPs correlated with high (up to 50%) relative abundance of genera *Bifidobacterium* and *Lactobacillus*, as compared to the IMMP blank group, in which less than 5% of all reads belonged to these taxa. This prebiotic effect was especially strong in IMMP‐27, IMMP‐94, and IMMP‐96 after 24 h of incubation, at which time the fermentation of α‐(1→6) linked glucosyl residues was predominant. The increase in relative abundance of *Bifidobacterium* and *Lactobacillus* was specific to the presence of IMMPs and was not observed in the IMMP blank, indicating that the fermentation of IMMPs promoted the growth of *Bifidobacterium* and *Lactobacillus* (**Table** 2).

**Table 2:** Genus level taxa with significantly different relative abundance in combined IMMP groups at 24 and 48 h of incubation as compared to IMMP blank groups at 24 and 48 h using Kruskal–Wallis analysis

				Average RA	
Taxon	P	FDR_P	Bonferroni_P	IMMP	Blank
*g__Bifidobacterium*	0.00001	0.00006	0.00029	0.263	0.008
*g__Escherichia–Shigella*	0.01045	0.02438	0.43878	0.184	0.319
*g__Lactobacillus*	0.00001	0.00006	0.00029	0.096	0.005
*g__Parabacteroides*	0.00922	0.02278	0.38733	0.023	0.042
*g__Sutterella*	0.00023	0.00109	0.00981	0.014	0.030
*f__Bifidobacteriaceae_g__g*	0.00000	0.00006	0.00017	0.006	0.000
*o__Bifidobacteriales_g__g*	0.00479	0.01341	0.20118	0.002	0.000
*g__Parasutterella*	0.00000	0.00005	0.00005	0.000	0.013
*g__Bilophila*	0.00011	0.00060	0.00476	0.000	0.078
*g__Pseudobutyrivibrio*	0.00164	0.00492	0.06895	0.000	0.005
*g__Alistipes*	0.00164	0.00492	0.06895	0.000	0.005
*g__Eggerthella*	0.00045	0.00171	0.01879	0.000	0.003
*f__Lachnospiraceae_g__g*	0.00001	0.00006	0.00024	0.000	0.003
*g__Ruminococcus*	0.00003	0.00016	0.00111	0.000	0.003
*g__Subdoligranulum*	0.00003	0.00016	0.00111	0.000	0.002
*g__Butyricimonas*	0.00045	0.00171	0.01879	0.000	0.002
*f__Ruminococcaceae_g__g*	0.00164	0.00492	0.06895	0.000	0.001
*g__Methanobrevibacter*	0.00565	0.01484	0.23749	0.000	0.001

For IMMP‐96 and IMMP‐dig27, the relative abundance of *Bifidobacterium* remained very low in the first 12 h (2–5%), then increased rapidly to a high level at 24 h (27% for IMMP‐96; 9% for IMMP‐dig27). From 24 to 48 h, *Bifidobacterium* relative abundance continued to largely increase for IMMP‐96 (44%), whereas it only slightly increased for IMMP‐dig27 (11%). The growth pattern of bifidobacteria was in line with the degradation pattern of IMMPs which consisted mostly of α‐1,6‐linkages, as both started only after 12 h of fermentation. The highest increase in relative abundance of bifidobacteria occurred from 12 to 24 h, where IMMP polysaccharides were degraded into α‐1,6‐linked IMOs with DP of 7 to over 20. The growth pattern of bifidobacteria also agreed with the formation of SCFAs, as shown previously. The formation of SCFAs during fermentation contributes to acidification of the colonic lumen.^4^ A lower pH in the colon is favorable for bifidobacteria and lactic acid bacteria, while impeding the overgrowth of more pH‐sensitive pathogenic bacteria.^2^, ^42^ For IMMP‐94 and IMMP‐27, microbiota composition at 6 and 12 h was not analyzed, due to a scarcity of the fermentation digest. Both IMMPs showed high levels of *Bifidobacterium* at 24 h (29% for IMMP‐94; 25% for IMMP‐27), and at 48 h the relative abundance of this genus remained almost the same for IMMP‐94 (30%) but slightly decreased for IMMP‐27 (21%).

The observed changes in relative abundance of *Lactobacillus* differed among different IMMPs, with the strongest increase observed for IMMP‐94 and IMMP‐27 at 24 and 48 h, whereas the increase in relative abundance was weaker with IMMP‐96 and IMMP‐dig27. In the presence of IMMP‐96, there was a rapid increase in the relative abundance at 6 h, followed by a gradual decline at later time points, whereas with IMMP‐dig27 the relative abundance of this genus showed a steady increase with time. The more pronounced increase in relative abundance of *Lactobacillus* during the fermentation of IMMP‐94 and IMMP‐27 as compared to the other two substrates was in line with the higher level of lactic acid produced during fermentation of IMMP‐94 and IMMP‐27 (Figures 4 and 7). In addition, there was an increase in the relative abundance of genera *Enterococcus* and *Parabacteroides* during the fermentation of IMMP‐dig27 and with the IMMP blank, but not for the other IMMPs (Figure 10B), a result which we cannot explain in a straightforward way. However, there is a growing evidence suggesting that metabolic webs and complex polysaccharide utilization networks exist between different members of intestinal microbiota, with different species specializing to utilize different polysaccharides, expanding the number and types of glycoside hydrolase produced in the presence of a competitor, or acting as producers or recipients of the polysaccharide breakdown products.^43^, ^44^

We observed a high accumulation of succinate during the in vitro fermentation of all IMMPs. This might be due to activity of *Bacteroides* which in the gut can use CO_2_ to reduce formate to succinate to generate ATP in a primitive electron transport chain.^43^ Succinate is then excreted as an end product and can be utilized by secondary fermenters, or it can be further converted by *Bacteroides* to propionate if the CO_2_ is limiting. In fact, the ability to convert succinate to propionate has been described for both Bacteroidetes and Veillonellaceae.^31^ In auxotrophic *Bacteroides* spp. this conversion of succinate to propionate is modulated by the availability of vitamin B12, which in the gut is produced by certain members of Firmicutes and Actinobacteria.^45^ Thus, the accumulation of succinic acid in our experiment could be, among others, a result of high CO_2_ levels, vitamin B12 limitation, or might be linked to the absence of members of the family Veillonellaceae, which were not detected in our microbiota composition analysis (Figure 10B).

## 4 Conclusions

IMMPs showed delayed and slow‐fermenting behavior compared to other prebiotics during their in vitro fermentation by a human fecal inoculum. Measurable production of enzymes targeting α‐1,6‐linked glucose was detected after 12 h of incubation. The presence of α‐(1→4) linked glucosyl linkages in the IMMPs further postponed the bacterial utilization of α‐(1→6) linked glucosyl residues, suggesting that when available, the α‐(1→4) linked glucosyl residues are preferentially used by the fecal microbiota. We also found that α‐(1→6) linked glucose oligomers with lower DP were preferentially used, as compared to those with higher DP. Organic acids were produced at high total amounts during IMMPs’ fermentation, with acetic acid and succinic acid being the predominant metabolites in all incubations. The HPAEC chromatograms and enzyme production analysis showed that the polysaccharide fraction of IMMPs was degraded mainly by extracellular enzymes into α‐(1→6) linked IMOs, among which the IMOs with DP lower than seven might be transported into microbial cells and further degraded by cytoplasmic enzymes. Fermentation of IMMPs led to the increase in diversity and evenness of bacterial communities, and promoted the increase in relative abundance especially of genera *Bifidobacterium* and *Lactobacillus*, lending a strong support for the prebiotic potential of these fibers.

## Conflict of Interest

The authors declare no conflict of interests.

## Supporting information

Supporting Information

## References

[1] G. R. Gibson , R. Hutkins , M. E. Sanders , S. L. Prescott , R. A. Reimer , S. J. Salminen , K. Scott , C. Stanton , K. S. Swanson , P. D. Cani , K. Verbeke , G. Reid , Nat. Rev. Gastroenterol. Hepatol. 2017, 14, 491. 28611480 10.1038/nrgastro.2017.75

[2] G. R. Gibson , Clin. Nutr. Suppl. 2004, 1, 25.

[3] K. P. Scott , J. C. Martin , S. H. Duncan , H. J. Flint , FEMS Microbiol. Ecol. 2014, 87, 30. 23909466 10.1111/1574-6941.12186

[4] J. Verspreet , B. Damen , W. F. Broekaert , K. Verbeke , J. A. Delcour , C. M. Courtin , Annu. Rev. Food Sci. Technol. 2016, 7, 81315. 10.1146/annurev-food-081315-032749 26735801

[5] J. M. Dobruchowska , G. J. Gerwig , S. Kralj , P. Grijpstra , H. Leemhuis , L. Dijkhuizen , J. P. Kamerling , Glycobiology 2012, 22, 517. 22138321 10.1093/glycob/cwr167

[6] H. Leemhuis , J. M. Dobruchowska , M. Ebbelaar , F. Faber , P. L. Buwalda , M. J. E. C. van der Maarel , J. P. Kamerling , L. Dijkhuizen , J. Agric. Food Chem. 2014, 62, 12034. 25412115 10.1021/jf503970a

[7] D. Goffin , N. Delzenne , C. Blecker , E. Hanon , C. Deroanne , M. Paquot , Crit. Rev. Food Sci. Nutr. 2011, 51, 394. 21491266 10.1080/10408391003628955

[8] J. Kaulpiboon , P. Rudeekulthamrong , S. Watanasatitarpa , K. Ito , P. Pongsawasdi , J. Mol. Catal. B Enzym. 2015, 120, 127.

[9] A. Ketabi , L. A. Dieleman , M. G. Gänzle , J. Appl. Microbiol. 2011, 110, 1297. 21338450 10.1111/j.1365-2672.2011.04984.x

[10] C. E. Rycroft , M. R. Jones , G. R. Gibson , R. A. Rastall , J. Appl. Microbiol. 2001, 91, 878. 11722666 10.1046/j.1365-2672.2001.01446.x

[11] E. Olano‐Martin , K. C. Mountzouris , G. R. Gibson , R. A. Rastall , Br. J. Nutr. 2000, 83, 247. 10884713 10.1017/s0007114500000325

[12] B. A. Ashwar , A. Gani , A. Shah , I. A. Wani , F. A. Masoodi , Starch/Staerke 2016, 68, 287.

[13] M. C. Jonathan , D. Haenen , C. Souza Da Silva , G. Bosch , H. A. Schols , H. Gruppen , Carbohydr. Polym. 2013, 93, 232. 23465924 10.1016/j.carbpol.2012.06.057

[14] C. Rösch , K. Venema , H. Gruppen , H. A. Schols , Bioact. Carbohydrates Diet. Fibre 2015, 6, 46.

[15] U. S. Ramasamy , H. A. Schols , H. Gruppen , Bioact. Carbohydrates Diet. Fibre 2014, 4, 115.

[16] P. H. van der Zaal , H. A. Schols , J. H. Bitter , P. L. Buwalda , Carbohydr. Polym. 2018, 185, 179. 29421055 10.1016/j.carbpol.2017.11.072

[17] Y. Bai , R. M. van der Kaaij , H. Leemhuis , T. Pijning , S. S. van Leeuwen , Z. Jin , L. Dijkhuizen , Appl. Environ. Microbiol. 2015, 81, 7223. 26253678 10.1128/AEM.01860-15 PMC4579422

[18] M. Dubois , K. A. Gilles , J. K. Hamilton , P. A. Rebers , F. Smith , Anal. Chem. 1956, 28, 350.

[19] A. K. Saha , C. F. Brewer , Carbohydr. Res. 1994, 254, 157. 8180982 10.1016/0008-6215(94)84249-3

[20] M. Aguirre , J. Ramiro‐Garcia , M. E. Koenen , K. Venema , J. Microbiol. Methods 2014, 107, 1. 25194233 10.1016/j.mimet.2014.08.022

[21] M. Minekus , M. Smeets‐Peeters , A. Bernalier , S. Marol‐Bonnin , R. Havenaar , P. Marteau , M. Alric , G. Fonty , J. H. J. Huis in't Veld , Appl. Microbiol. Biotechnol. 1999, 53, 108. 10645630 10.1007/s002530051622

[22] S. E. Ladirat , H. A. Schols , A. Nauta , M. H. C. Schoterman , F. H. J. Schuren , H. Gruppen , Bioact. Carbohydrates Diet. Fibre 2014, 3, 59.

[23] J. Ramiro‐Garcia , G. D. A. Hermes , C. Giatsis , D. Sipkema , E. G. Zoetendal , P. J. Schaap , H. Smidt , F1000Research 2016, 5, 1791. 10.12688/f1000research.9227.1 PMC6419982 30918626

[24] J. G. Caporaso , J. Kuczynski , J. Stombaugh , K. Bittinger , F. D. Bushman , E. K. Costello , N. Fierer , A. G. Pena , J. K. Goodrich , J. I. Gordon , G. A. Huttley , S. T. Kelley , D. Knights , J. E. Koenig , R. E. Ley , C. A. Lozupone , D. McDonald , B. D. Muegge , M. Pirrung , J. Reeder , J. R. Sevinsky , P. J. Turnbaugh , W. A. Walters , J. Widmann , T. Yatsunenko , J. Zaneveld , R. Knight , Nat. Methods 2010, 7, 335. 20383131 10.1038/nmeth.f.303 PMC3156573

[25] J. Kuczynski , J. Stombaugh , W. A. Walters , A. Gonzalez , J. G. Caporaso , R. Knight , Curr. Protoc. Microbiol. 2011, 36, 10.7.1. 10.1002/9780471729259.mc01e05s27 PMC4477843 23184592

[26] M. J. E. C. Van Der Maarel , I. Capron , G. J. W. Euverink , H. T. Bos , T. Kaper , D. J. Binnema , P. A. M. Steeneken , Starch/Staerke 2005, 57, 465.

[27] Q. Wu , X. Pi , W. Liu , H. Chen , Y. Yin , H. D. Yu , X. Wang , L. Zhu , Anaerobe 2017, 48, 206. 28882708 10.1016/j.anaerobe.2017.08.016

[28] S. Macfarlane , G. T. Macfarlane , Proc. Nutr. Soc. 2003, 62, 67. 12740060 10.1079/PNS2002207

[29] Y. Watanabe , F. Nagai , M. Morotomi , Appl. Environ. Microbiol. 2012, 78, 511. 22081579 10.1128/AEM.06035-11 PMC3255759

[30] N. Reichardt , S. H. Duncan , P. Young , A. Belenguer , C. M. Leitch , K. P. Scott , H. J. Flint , P. Louis , ISME J. 2014, 8, 1323. 24553467 10.1038/ismej.2014.14 PMC4030238

[31] H. J. Flint , K. P. Scott , P. Louis , S. H. Duncan , Nat. Rev. Gastroenterol. Hepatol. 2012, 9, 577. 22945443 10.1038/nrgastro.2012.156

[32] A. Biswas , R. L. Shogren , S. Kim , J. L. Willett , Carbohydr. Polym. 2006, 64, 484.

[33] S. S. Sahota , P. M. Bramley , I. S. Menzies , J. Gen. Microbiol. 1982, 128, 319. 6804597 10.1099/00221287-128-2-319

[34] C. Demigné , H. Jacobs , C. Moundras , M.‐J. Davicco , M.‐N. Horcajada , A. Bernalier , V. Coxam , Eur. J. Nutr. 2008, 47, 366. 18779917 10.1007/s00394-008-0736-5

[35] D. Dimitrovski , E. Velickova , M. Dimitrovska , T. Langerholc , E. Winkelhausen , J. Food Sci. Technol. 2016, 53, 766. 26787997 10.1007/s13197-015-2064-0 PMC4711484

[36] A. G. M. Leijdekkers , M. Aguirre , K. Venema , G. Bosch , H. Gruppen , H. A. Schols , J. Agric. Food Chem. 2014, 62, 1079. 24437353 10.1021/jf4049676

[37] J. M. W. Wong , R. de Souza , C. W. C. Kendall , A. Emam , D. J. A. Jenkins , J. Clin. Gastroenterol. 2006, 40, 235. 16633129 10.1097/00004836-200603000-00015

[38] T. Kohmoto , F. Fukui , H. Takaku , Y. Machida , M. Arai , T. Mitsuoka , Bifidobact. Microflora 1988, 7, 61.

[39] T. Kohmoto , F. Fukui , H. Takaku , T. Mitsuoka , Agric. Biol. Chem. 1991, 55, 2157.

[40] G. T. Macfarlane , H. N. Englyst , J. Appl. Bacteriol. 1986, 60, 195. 2423494 10.1111/j.1365-2672.1986.tb01073.x

[41] T. S. Manning , G. R. Gibson , Best Pract. Res. Clin. Gastroenterol. 2004, 18, 287. 15123070 10.1016/j.bpg.2003.10.008

[42] P. Reviews , D. L. Topping , P. M. Clifton , Physiol. Rev. 2001, 81, 1031. 11427691 10.1152/physrev.2001.81.3.1031

[43] M. A. Fischbach , J. L. Sonnenburg , Cell Host Microbe 2011, 10, 336. 22018234 10.1016/j.chom.2011.10.002 PMC3225337

[44] M. S. Datta , J. Gore , Curr. Biol. 2014, 24, R33. 24405677 10.1016/j.cub.2013.11.023

[45] A. L. Goodman , N. P. McNulty , Y. Zhao , D. Leip , R. D. Mitra , C. A. Lozupone , R. Knight , J. I. Gordon , Cell Host Microbe 2009, 6, 279. 19748469 10.1016/j.chom.2009.08.003 PMC2895552

