# Supplementary material for: In Vitro Fermentation Behavior of Isomalto/Malto‐Polysaccharides Using Human Fecal Inoculum Indicates Prebiotic Potential
Source: Mol Nutr Food Res. 2018 May 28;62(12):1800232. doi: 10.1002/mnfr.201800232 (PMC6033187; doi:10.1002/mnfr.201800232)

Supporting Information Table S1. Relative abundance of taxa detected during *in vitro* fermentation of IMMPs with human faecal inoculum at different time points at genus level. Where no classification was available at genus level, higher taxonomic level was used and the unclassified genus is denoted as g_g.


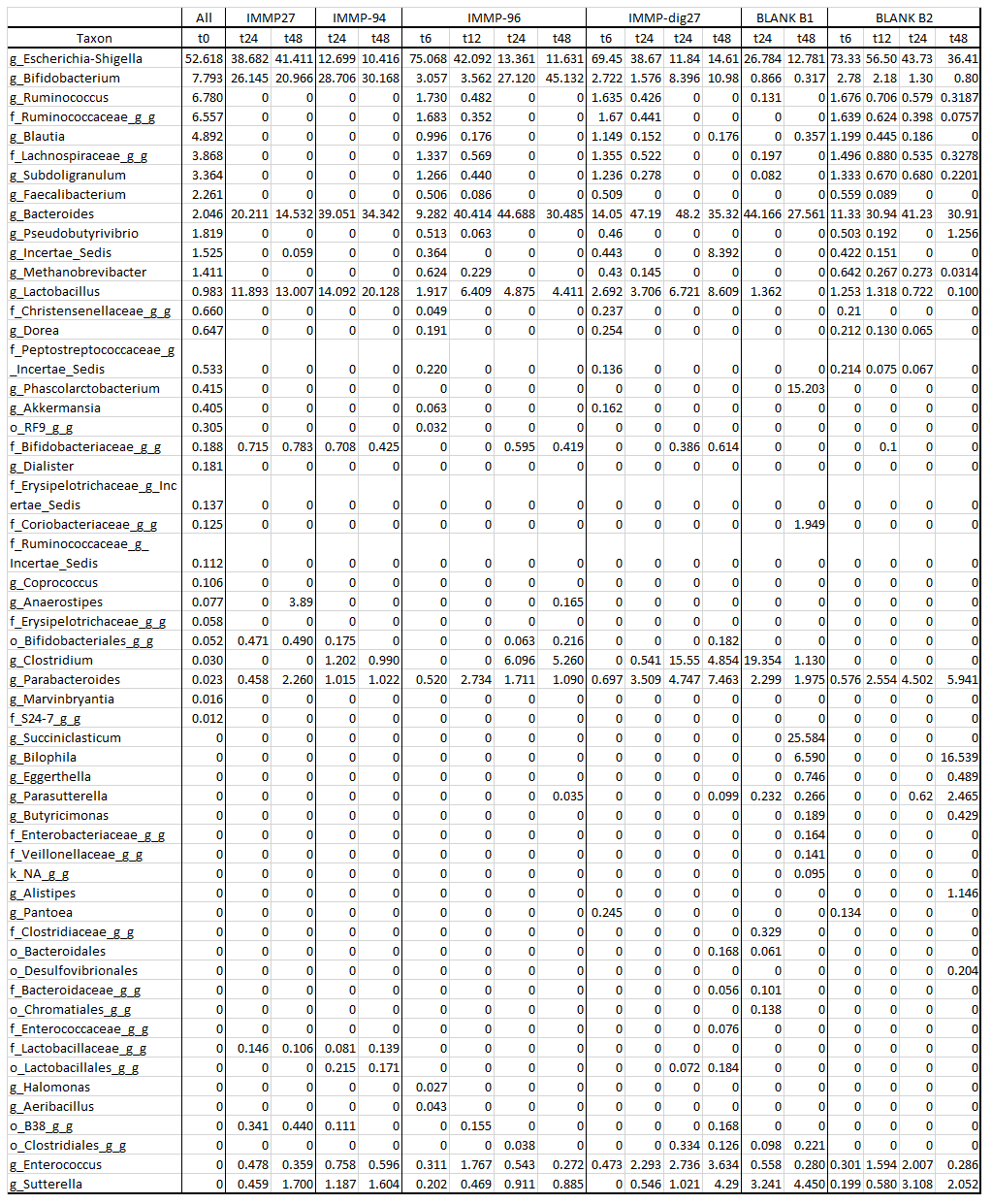

Supplement: Supplementary file 1 — Supporting Information [file MNFR-62-na-s001.docx]
